# Update of the clinical guideline for hypertension diagnosis and treatment in Iran

**DOI:** 10.1186/s40885-024-00269-6

**Published:** 2024-06-01

**Authors:** Fahimeh Bagherikholenjani, Shahla Shahidi, Alireza Khosravi, Asieh Mansouri, Vahid Ashoorion, Nizal Sarrafzadegan, Guideline Updating Group (GUG), Mansour Siavash, Shahrzad Shahidi, Fariborz Khorvash, Masoumeh Sadeghi, Hossein Farshidi, Ahmadreza Assareh, Davood Shafiei, Masoumeh Jorjani, Shirinsadat Badri, Valiollah Hajhashemi, Ramesh Hoseinkhani, Mojgan Mortazavi, Mojdeh Ghabaei, Somayeh Khanjani, Elham Hashemi, Bahar Dehghan, Majid Davari, Behzad Fatemi, Noushin Mohammadifard, Majid Ghayour Mobarhan, Maryam Eghbali babadi, Alireza Ahmadi, Razieh Hassannejad, Fereidoun Noohi, Steering Committee, Maryam Kheiri, Mosa Tabatabaeilotfi, Sanaz Bakhshandeh, Azadeh Haghighi, Systematics Review Group (SRG), Marjan Mansourian, Ziba Farajzadegan, Hale Ashraf, Negar Omidi, Negah Tavakolifard, Mahasti Alizade, Golnaz Vaseghi, External Review Group (ERG), Ebrahim Nematipour, Samad Ghaffari, Mojgan Sanjari, Mahmoud Mohammadzade Shabestari, Maryam Heidarpour

**Affiliations:** 1 Isfahan Cardiovascular Research Center, Cardiovascular Research Institute, Isfahan University of Medical Sciences, Isfahan, Iran; 2 Hypertension Research Center, Cardiovascular Research Institute, Isfahan University of Medical Sciences, Isfahan, Iran; 3 Iranian Network of Cardiovascular Research, Tehran, Iran; 4 Humber River Hospital, Toronto, Canada

**Keywords:** Hypertension, Blood Pressure, Clinical practice Guideline, Updating, Diagnosis, Treatment

## Abstract

**Background:**

This article introduces the updated version of the Iranian guideline for the diagnosis and treatment of hypertension in adults. The initial version of the national guideline was developed in 2011 and updated in 2014. Among the reasons necessitating the update of this guideline were the passage of time, the incompleteness of the scopes, the limitation of the target group, and more important is the request of the ministry of health in Iran.

**Method:**

The members of the guideline updating group, after reviewing the original version and the new evidence, prepared 10 clinical questions regarding hypertension, and based on the evidence found from the latest scientific documents, provided recommendations or suggestions to answer these questions.

**Result:**

According to the updated guideline, the threshold for office prehypertension diagnosis should be considered the systolic blood pressure (SBP) of 130-139 mmHg and/or the diastolic blood pressure (DBP) of 80-89 mmHg, and in adults under 75 years of age without comorbidities, the threshold for office hypertension diagnosis should be SBP ≥ 140 mmHg and or DBP ≥ 90 mmHg.

The goal of treatment in adults who lack comorbidities and risk factors is SBP < 140 mmHg and DBP < 90 mmHg. The first-line treatment recommended in people with prehypertension is lifestyle modification, while for those with hypertension, pharmacotherapy along with lifestyle modification. The threshold to start drug therapy is determined at SBP ≥ 140 mmHg and or DBP ≥ 90 mmHg, and the first-line treatment is considered a drug or a combined pill of antihypertensive drugs, including ACEIs, ARBs, thiazide and thiazide-like agents, or CCBs.

At the beginning of the pharmacotherapy, the Guideline Updating Group members suggested studying serum electrolytes, creatinine, lipid profile, fasting sugar, urinalysis, and an electrocardiogram. Regarding the visit intervals, monthly visits are suggested at the beginning of the treatment or in case of any change in the type or dosage of the drug until achieving the treatment goal, followed by every 3-to-6-month visits. Moreover, to reduce further complications, it was suggested that healthcare unit employees use telehealth strategies.

**Conclusions:**

In this guideline, specific recommendations and suggestions have been presented for adults and subgroups like older people or those with cardiovascular disease, diabetes mellitus, chronic kidney disease, and COVID-19.

## Background

According to the conducted research, the volume of scientific information is increasing exponentially, and approximately 75 clinical trials and 11 systematic reviews are published daily [1]. Dealing with this growing amount of information and utilizing it to make the best clinical decisions requires keeping the available resources, especially clinical guidelines, up-to-date [2]. Clinical guidelines are a set of instructions aimed at optimizing patient care and are prepared based on the systematic review of available resources and taking into account the advantages and disadvantages of other care options [3]. Considering the importance of clinical guidelines, updating them is essential to guarantee the validity of the guidelines and maintain their benefits for patients, healthcare providers, and other beneficiaries [4].

There is no single and uniform opinion about the optimal time interval for updating clinical guidelines. Some resources have suggested this interval be between 3 and 5 years [5, 6]. On the other hand, updating guidelines by adopting a time-based approach is not necessarily a suitable method for all clinical guidelines due to the fact that the rate of changes in scientific resources is not the same. According to some researchers, the priority-setting approach (updating by taking into account the priority of updating clinical guidelines whose related documents and scientific evidence have changed sufficiently) is a more efficient method than the time-based method; however, this method requires active monitoring of scientific resources [7].

One of the most important health problems, the management of which is highly important due to being based on accurate and up-to-date instructions, is hypertension [8]. Essential hypertension is one of the main causes of disability and death and has a proven role in causing cardiovascular events [9]; nevertheless, it is possible to control it. Considering that one of the known obstacles in society regarding the management of this disease is related to the physicians' lack of knowledge and awareness or their failure to follow the available guideline for the control and care of this disease [10], it seems necessary to offer physicians a set of clinical solutions (which naturally cause the least harm and damage to the patients) based on the latest scientific evidence.

In different countries, various clinical strategies are developed for the prevention, control, and treatment of blood pressure that are updated at regular intervals. Some of the most important clinical strategies include the guideline for the prevention, detection, evaluation, and management of high blood pressure in adults [10] presented by the American Heart Association, guideline for the management of arterial hypertension presented by the European Society of Cardiology and the European Society of Hypertension [11], guideline for diagnosis, risk assessment, prevention, and the treatment of hypertension in adults provided by the Canadian Hypertension Education Program [12], and the guideline for hypertension in adults provided by the National Institute for Health and Healthcare Excellence in England [13].

In Iran, with the aim of providing an evidence-based approach for the prevention, treatment, and control of hypertension based on existing international solutions, and considering the results of regional research, Iran’s social situation, and its healthcare needs, the first version of this guideline was compiled in 2011 and updated in 2014 with the cooperation of professors from various Iranian universities and organizations along with foreign experts who were all active in preparing guidelines for their countries or regions.

In the initial version of the guideline, it was decided that it should be revised every 3 years [14]. Since maintaining the dynamics of clinical guidelines is undeniable due to the changes that occur over time in medical evidence (especially in diagnostic and treatment methods, the incidence and prevalence of diseases, and patients' lifestyle and their conditions), as well as taking into account an important official request of the Office of Health Technology Assessment, Standardization, and Tariff of the Iranian Ministry of Health (MOH) in this regard, and considering some of the limitations of the previous versions and the extent of novel developments in the management and treatment of hypertension, the MOH decided to update the guideline for the diagnosis and treatment of hypertension in Iran. As a result, important information sources in accordance with the latest scientific documents accepted by prominent international scientific communities regarding the diagnosis, and treatment of hypertension have been developed based target groups.

## Methods

The process of updating the guideline was initiated following discussion of the members of the Steering Committee, and establishing the Guideline Updating Group (GUG). At the same stage, the External Review Group (ERG) was determined and the conflicts of interest of all groups were carefully evaluated. The GUG consisted of 38 experts in related fields from various universities of medical sciences, members of the national network of research in Iran, research institutes, research centers, and scientific associations, as well as the staff involved in providing relevant services. The systematic review group (SRG), who were independent of the GUG, were selected through a call and their conflicts of interest were investigated. The process of updating this guideline was accomplished in three phases. In the first stage, the scope of the guideline was determined considering the initial version of the guideline, and questions in the field of scope were collected to develop the Population, Intervention, Comparison, and Outcomes (PICOs). In the second phase, systematic review was done. The PICOs were sent to the SR group who did extensive review and grading to develop the evidence and in the third phase recommendations and suggestions were prepared. Finally, guideline was written and the corresponding algorithm was drawn.

The first and third phases were conducted during 23 online meetings of the GUG with the cooperation of each member of this group, while the second phase was done by the systematic review group.

### Participating groups in updating the guideline

The participating groups in developing this guideline consist of:

- *Steering Committee (SC)*: This committee included the project executive, main collaborators, and the officials of the Office of Health Technology Assessment, Standardization, and Tariff of the Ministry of Health. It was in charge of selecting the members of other groups, holding the meetings and implementing the steps of updating the guideline, monitoring the time and finalizing the guideline.
- *Guideline Updating Group (GUG)*: This group consisted of experts from national universities of medical sciences and related scientific societies or research institutes. Members of the National Network of CVD Research participated too. The participants were specialists, namely internists, cardiologists, endocrinologists, nephrologists, neurologists, pediatricians, obstetricians and gynecologists, nutritionists, epidemiologists, and pharmacologists, Health economists as well as general physicians, nurses, and staff working in the healthcare system. These individuals were selected based on their expertise, and interest from all over the country. A methodologist facilitated the GUG group meetings.
- *Systematic Review Group (SRG)*: The members of this group were independent from the GUG members and were experts in the field of systematic review and meta-analysis. This group was responsible for searching, developing and summarizing the evidence.
- *External Review Group (ERG)*: It included a number of experts in various fields related to the subject as well as influential people in policymaking in this field that were all responsible to evaluate the updated guideline. All were external to the GUG or other groups in developing this guideline.

### Declaration of conflicts of interest

To identify the types of conflicts of interest (e.g., finance, work, research, and consultancy), the standard conflict of interest approved by the Ministry of Health of Iran was signed by all members of the groups involved in updating the guideline. There were no cases of conflict of interest in the completed forms; however, according to prior planning, it was decided that if any conflict of interest was identified, while maintaining the confidentiality of information, the cases would be managed by the SC and then possible measures.

### Scope of guidelines and questions

The scope of the guideline included the functional area, the target group (i.e., individuals who might be affected by the recommendations), and the results obtained from the guideline. The *functional scope of the last updated guideline*, according to the GUG members, did not deal with diagnosis.

The *target group of the guideline*, according to the decision of the GUG group, was initially people with hypertension and included subgroups of children, pregnant women, the elderly, individuals with cardiovascular diseases (CVDs), diabetes mellitus (DM), chronic kidney disease (CKD), and COVID-19. Nevertheless, at the end of the first phase of the process, considering the vast extent and spectrum of blood pressure in children and pregnant women, the GUG agreed that these two subgroups be excluded, and it was decided that separate guideline be developed for these two subgroups in the future. As a result, the target group of this guideline was considered as male and female adults (18 years old and above) with hypertension.

To determine and rank the outcomes, the members of the SC group prepared an initial list of outcomes proposed in the initial version of the hypertension guideline in 2011 and other outcomes in the latest published hypertension guideline at global level. Afterward, the members of the GUG were asked to review these outcomes and provide their proposed outcomes via an electronic form sent to them. following, the lists of primary and proposed outcomes were reviewed by GUG members in a meeting and the final outcomes were determined and ranked.

**Fig. 1:**
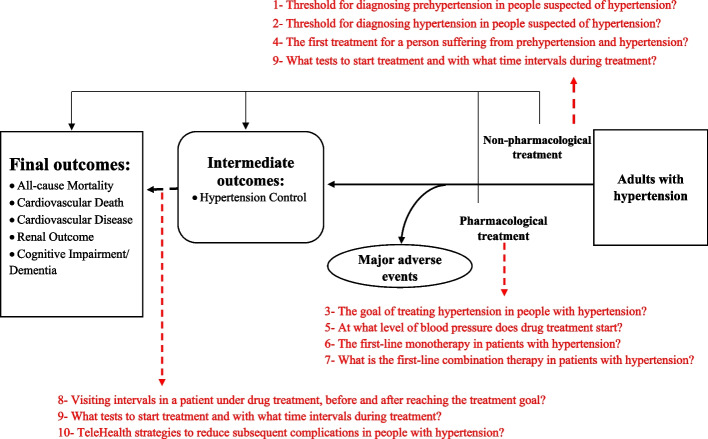
Analytical framework of hypertension management in adults

Next, during another process, primary or clinical questions and PICO questions were extracted after removing unrelated and repetitive items and merging similar ones, eventually, ten PICO questions were finalized and provided to the SR group. The members of the working group also created an analytical framework (Fig. 1) that showed the impact of interventions on intermediate and final outcomes and specified the order of the 10 PICO questions for better visualization and placing them along the path of patient management. To determine the values and preferences of patients with hypertension, qualitative research was performed in the form of two sessions for patients and physicians. A session of focus group discussion (FGD) that was held with the participation of 7 patients suffering from different levels of hypertension. During this session, the participants' preferences for diagnosis, medicinal and non- medicinal therapy (i.e., lifestyle), and required training were extracted. In addition to that, the values, preferences, and views of general practitioners regarding the problems of patients with hypertension were also determined during a virtual FGD session with the participation of 5 general practitioners who had a clinical practice in the field of hypertension. Their preferences were extracted in four axes consisting of diagnosis, treatment, use and expectation of the Iranian hypertension guideline. The values and preferences of physicians and patients were considered in the design of PICO questions and in later phases of developing this guideline (Fig. 1).

### Search for evidence

All PICO questions were submitted to the SR group. The SR group performed an extensive systematic search for each PICO question in PubMed, Embase, and Cochrane Library databases with a time limit for the last 6 years. At first, an Umbrella Systematic Review was conducted on all previous systematic reviews. In this way, the latest information related to new systematic reviews was found, and since there was no new systematic review for one of the PICO questions, a new one was conducted. Moreover, the evidence used in preparing the recent hypertension guideline was reviewed. The identified systematic reviews were evaluated in terms of their up-to-datedness, the degree of compliance with the PICO questions, the assessment of their methodology by the AMSTAR tool, the provision of sufficient information to evaluate the certainty of the evidence, and reporting evidence on different outcomes and in the subgroups of the PICOs; subsequently, the most appropriate ones were selected.

### Evidence to recommendations

After searching for scientific evidence through a systematic review, certainty in and quality of evidence were rated by the standard GRADE method. In this process, the risk of bias, imprecision, indirectness, and inconsistency were investigated. Additionally, another overview was conducted by the SC to examine other decision-making criteria in the framework of the Evidence to Decision tables, and the evidence related to the criteria of certainty, patients' values and preferences, health benefits and harms, resources, costs, acceptability, the feasibility of implementation, and health equity indicators were discussed.

### Compilation of recommendations and suggestions

To compile the recommendations, 9 two-hour online meetings were held with the members of the GUG group. In each meeting, one or two PICO question was introduced and then its related evidence provided by the SR group was presented in the form of a GRADE evidence profile by the head of SR group. Subsequently, the members of the GUG group, with the guidance of the methodologists, completed the evidence-to-decision tables.

The recommendations of this guideline were divided into two groups based on the strength of the evidence in the recommendation. The strength of recommendations indicated the extent to which the GUG was confident that the desirable effects of the recommendations (e.g., beneficial health outcomes) outweigh the potential undesirable effects (e.g., side effects). The classification of the recommendations in this hypertension guideline was as follows:

1. Strong recommendation: A recommendation for which the GUG was confident that the favorable effects of adhering to it would outweigh its adverse effects.
2. Suggestion or Conditional Recommendation: A recommendation for which there was greater uncertainty regarding the quality of the evidence, the balance of benefits and harms, values and preferences, and the use of resources; however, the GUG concluded that the positive effects of adhering to it probably outweighed its negative effects. These cases were presented as "Suggestions". In some cases, when there was extensive discussion on the evidence, a consensus of GUG members on the recommendation was reached

### External evaluation

The initial report of the guideline along with the AGREE Reporting Checklist was provided to the ERG (an independent group from the GUG) as a guideline evaluation tool to evaluate the draft guideline in terms of validity, reliability, clarity, clinical applicability, clinical flexibility, and documentation. Then, based on their opinions and comments, the guideline was re-examined and some parts were modified. Moreover, to facilitate the use of the guideline, based on the recommendations, an algorithm was developed for the diagnosis and treatment of hypertension.

### Update time

The update time for the current guideline was set for the next 3 years.

## Results

According to the previous explanations, 36 recommendations and suggestions (18 recommendations and 18 suggestions) were developed. As for COVID-19 subgroup, the systematic review group did not find evidence of differences in the diagnosis and treatment of hypertension in covid-19 patients compared to the general population, so no specific recommendations or suggestions were provided in the field of covid-19. The recommendations and suggestions extracted for the PICO questions related to the updated guideline for the diagnosis, management, and treatment of hypertension along with the relevant documents and evidence are as follows:

**Box 1:** Recommendation for diagnosing prehypertension

1- It is recommended that the threshold of prehypertension diagnosis be considered as systolic blood pressure (SBP) of 130–139 mm Hg and/or diastolic blood pressure (DBP) of 80–89 mm Hg for BP in the office	

### Evidence and rationale

Studies show that prehypertension is an independent risk factor for CVDs [15]. It has been reported that prehypertension, especially in a high range (SBP 130- 139 mmHg and/or DBP 85–89 mmHg), is associated with increased risk of CVDs, coronary heart disease, myocardial infarction (MI), and stroke, and effective control of hypertension can prevent more than 10% of CVD cases.

The overall prevalence of prehypertension in Iran is 31.6% based on the results of a systematic review of 48 studies with 417,349 participants [16]. A meta-analysis performed on 29 clinical trial studies with 127,641 participants demonstrated that the identification of patients with prehypertension and their active treatment compared to placebo had led to a 7% reduction in the risk of the first outcomes of CV events, 14% decrease in stroke, and 10% decline in heart failure (HF) [15].

### Evidence-to-decision considerations

An asymptomatic patient may undervalue the importance of diagnosis and treatment of prehypertension unless they are convinced that there is a relationship between immediate side effects and potential long-term health gains [17]. The GUG assessed the resources and costs required for recommendations related to this question to be small. Cost-effectiveness analysis using the Systolic Blood Pressure Intervention Trial (SPRINT) has shown that although the treatment of high-risk/CVD patients with a baseline blood pressure of 130–139 mmHg is affordable, it does not help reduce costs, and the value of an intervention is associated with maintaining its effect for more than 5 years [18]. The effect of diagnosing prehypertension at the mentioned threshold on health inequalities was assessed as unclear because no evidence was found in this field. From the perspective of the panel members, the selected threshold was acceptable to the key beneficiaries and was implementable. In this regard, the results of our FGD with general practitioners demonstrated that they believe that different guidelines provided different numbers for the threshold of diagnosis. Therefore, it is necessary to provide a single diagnostic threshold for diagnosing prehypertension and hypertension in the country.

**Box 2:** Recommendation and suggestions for diagnosing hypertension

1- It is recommended that the threshold for diagnosing hypertension in adults under 75 years of age without comorbidities should be SBP of ≥ 140 mmHg and/or DBP of ≥ 90 mmHg using office BP measurement 2- It is suggested that the threshold for diagnosing hypertension in adults over 75 years of age should be SBP of ≥ 150 mmHg and/or DBP of ≥ 90 mmHg using office BP measurement 3- It is suggested that the threshold for diagnosing hypertension, in case of Ambulatory Blood Pressure Measurement (ABPM), should be considered SBP of ≥ 130 mmHg and/or DBP of ≥ 80 mmHg in an average whole day (24 h) 4- It is suggested that the threshold for diagnosing hypertension, in case of ABPM, should be considered SBP of ≥ 135 mmHg and/or DBP of ≥ 85 mmHg in the average day time or awake time 5- It is suggested that the threshold for diagnosing hypertension, in case of BP measurement at home, should be considered as SBP of ≥ 135 mmHg and/or DBP of ≥ 85 mmHg	

### Evidence and rationale

Despite strong evidence supporting the efficacy of antihypertensive therapy, major disagreements still exist, especially about the appropriate blood pressure threshold to start pharmacotherapy, target blood pressure during treatment, and treatment strategies, and these uncertainties have been reflected in the difference between clinical guidelines for the management of hypertension [10, 11, 19].

Based on the findings of a meta-analysis study of 48 randomized clinical trial studies, involving 344,716 participants with an average age of 65 years, a relative reduction in the risk of major adverse cardiovascular events (MACE) was seen in proportion to the reduction in BP level; accordingly, for every 5 mmHg reduction in SBP, the risk of MACE decreased by 10% [20].

In another meta-analysis of 61 prospective studies, the risk of CVD increased in a log-linear fashion from SBP levels of < 115 mmHg to < 180 mmHg and between DBP levels of < 75 mmHg to < 105 mmHg. Further analysis of the same study showed that a 20 mmHg increase in SBP and a 10 mmHg rise in DBP were associated with a doubling of mortality due to stroke and CVDs [21, 22].

Based on the results of studies, the overall prevalence of hypertension, awareness, treatment, and control in Iranian adult’s population were 20.4% (95% CI 16.5, 24.4; I2 = 99.9%), 49.3% (95% CI 44.8, 53.8; I2 = 98.5%), 44.8% (95% CI 28.3, 61.2; I2 = 99.9%), 37.4% (95% CI 29.0, 45.8; I2 = 99.3%), respectively [23, 24].

### Evidence-to-decision considerations

The expected benefits of lowering BP include reducing all course mortality, cardiovascular mortality, stroke, MI, and HF events [21, 22]. Regarding important damages related to the identification and diagnosis of hypertension (e.g., false positives, false negatives, anxiety, and psychological effects) and economic costs (e.g., time and loss of work or insurance), no evidence was found demonstrating that any of these damages are caused by the identification and diagnosis of hypertension; nevertheless, it is noteworthy that the absence of any evidence demonstrating harm does not guarantee that there is no harm [25].

The members of the GUG estimated the resources needed to diagnose blood pressure at the thresholds in the recommendation as medium and the required costs as low. In this regard, the results of American studies show that to improve the equitable control of high blood pressure in all people, health systems must evaluate and address the social needs related to the diagnosis, prevention, and successful control of blood pressure in each patient. Effectively addressing social factors influencing health requires investing in the infrastructure of health systems that are currently under pressure to become more cost-effective and value-oriented. This infrastructure requires a multifaceted approach [26, 27]. In terms of costs, people with hypertension in the United States incur an average of $2,000 more in annual healthcare costs than people without hypertension [28].

According to the members of the GUG, the established threshold for the diagnosis of hypertension was likely to reduce health inequalities. Barriers to accessing hypertension control care in low-income regions include the patient's low health literacy and limited resources [28]. However, it is possible to achieve fair and high rates of hypertension control among low-income and/or racially and ethnically diverse populations. The Million Hearts® Program publishes success stories of healthcare settings for underserved populations that have overcome access, economic, and cultural barriers, resulting in major improvements in hypertension control rates [29].

From the point of view of the panel members, the determined threshold was acceptable to the key beneficiaries and was implementable. The results of a qualitative study on several general practitioners revealed that they believed different guidelines provided different numbers for the diagnosis threshold, and there was a requirement to determine a single diagnostic threshold for the diagnosis of hypertension in the country [30].

**Box 3:** Recommendation and suggestion for treatment target of hypertension

1- It is recommended that the goal of treatment in adults under 75 years of age with hypertension and without comorbidities and risk factors should be SBP of < 140 mmHg and DBP of < 90 mmHg 2- It is suggested that the goal of treatment in elderly people over 75 years old should be SBP of < 150 mmHg and DBP of < 90 mmHg 3- It is recommended that in patients with CVDs, diabetes mellitus, a history of stroke, and CKD (who have albuminuria of ˃30 mg/g Cr), the target of treatment should be considered SBP of < 130 mmHg and DBP of < 80 mmHg	

### Evidence and rationale

In treating hypertension, physicians usually try to reach the target BP in patients. The target blood pressure is the blood pressure value below which there are favorable clinical benefits. The "the lower the better" approach, which has been of interest for many years in the treatment of hypertension, has been challenged over the past decade due to the lack of evidence from randomized trials supporting this approach. For this reason, the standard blood pressure target has been considered less than 140/90 mmHg for the general population of patients with hypertension during the past years.

The results of a systematic review with 38,688 participants with a mean follow-up period of 3.7 years revealed that lower goals did not reduce the overall rate of death and adverse events. This means that the benefits of lower blood pressure targets did not outweigh the harms compared to standard targets. Lower targets may reduce MI and congestive heart failure (CHF) but with low certainty for both outcomes [31].

In a systematic study on 8,221 adults with an average age of 74.8 years, higher BP targets (values less than 150/90 mmHg and less than 160/90 mmHg) were compared with lower targets (values less than 140/90 mmHg). The results of this study demonstrated that treatment for two different target blood pressures over 2 to 4 years failed to make a difference in any of the outcomes of death (from any cause), stroke, and MACE. However, the 95% confidence interval for these outcomes suggested that a lower target of BP might have a greater clinical benefit [32]. The findings of a systematic study with 42,134 participants showed that in people aged 75 years and older, strict SBP targets (120 mmHg and 130 mmHg) were associated with a significant reduction in the risk of all-cause mortality and cardiovascular mortality and heart failure, while higher SBP targets (150 mmHg and 160 mmHg) were linked to a lower risk of HF and stroke [33].

People with hypertension and established CVDs are at particular risk; in this regard, although decreasing blood pressure to a level lower than targets of people with high BP and no CVD may be beneficial and reduce cardiovascular mortality and morbidity, but it can increase the adverse effects. The results of a systematic review of 9,484 participants and a mean follow-up period of 3.7 years indicated that there was no significant difference in lower blood pressure targets for total mortality, cardiovascular deaths, total cardiovascular events, and serious adverse events [34].

### Evidence-to-decision consideration

Based on the results of our qualitative survey on patients with hypertension, patients believed the reason for treatment was to increase their life expectancy, prevent disease complications, and the fear of disability [30]; therefore, the goal of appropriate treatment should include the fulfillment of these expectations. The favorable effects of the lower blood pressure goal included a reduction in overall mortality, cardiovascular mortality, and stroke, while the reported adverse effect was an increase in side effects [35].

The members of the GUG evaluated the resources and costs required to achieve the mentioned treatment goals as average. Achieving good blood pressure control in health care in low-income countries is hindered by various obstructions and investing in the effective management of hypertension is often associated with various challenges. That is because dealing with chronic conditions like BP needs suitable policymaking along with a comprehensive and extensive implementation plan and documents that involve beneficiaries, health professionals, and resources to achieve this goal. The direct costs of health care related to the treatment of hypertension as well as risk factors and associated diseases, such as stroke, ischemic heart disease, and CHF, impose a significant financial burden. The results of a study by Howard et al. showed that the intensive management of uncontrolled hypertension was cost-effective compared to usual care [36].

Hypertension treatment leads to the prevention or reduction of mortality and cardiovascular events in the population and thus reduces health inequality. Uncontrolled high blood pressure may be more common in vulnerable populations. However, it is possible to achieve high and fair rates of hypertension control among vulnerable, low-income, and/or racially and ethnically diverse populations. Therefore, improving the treatment and control of hypertension through better treatment with the aim of reducing blood pressure can decrease long-term inequality [29]. In the opinion of the panel members, the determined treatment goals were acceptable and practical to the key beneficiaries.

**Box 4:** Recommendation and Suggestion for first line treatment of hypertension

1- It is recommended that lifestyle modification be considered the first therapeutic intervention in people with prehypertension, and in individuals with hypertension, both non-pharmacological and pharmacological treatment be considered General recommendations for lifestyle modification include the following: - Body mass index and waist circumference should be measured and it is recommended to keep both within a healthy range - The total daily intake of salt should be less than 5 g, the consumption of salty foods and adding salt during cooking should be limited, and using salt shaker should be avoided - Diet should contain fruits and vegetables, low-fat dairy products, whole grains, fish, poultry, vegetable protein, such as beans and oil nuts that are rich in magnesium, potassium, calcium, and fiber. In the diet of these people, saturated and trans fats, meat, sweets, and soft b drinks should be reduced - The use of potassium, calcium, and magnesium supplements is not recommended for the treatment of hypertension - Adults should perform at least 150–300 min of moderate-intensity aerobic physical activity or at least 75–150 min of vigorous-intensity aerobic physical activity in a week, or an equivalent combination of moderate-intensity and vigorous-intensity activity - Adults should do at least two days a week of moderate- or high-intensity activities that engage all major muscle groups - Any type of tobacco (e.g., cigarettes and hookah) and alcohol should be refrained in any amount, and in case of previous use, it should be stopped 2- It is suggested to consider stress coping methods, such as relaxation and yoga, as an intervention 3- It is suggested that people suffering from prehypertension and hypertension not to be exposed to polluted air 4-It is suggested that people suffering from prehypertension and hypertension and their families be educated on how to measure and monitor blood pressure as well as on adherence to drug treatment and lifestyle modification	

### Evidence and rationale

Choosing a healthy lifestyle can prevent or delay the onset of hypertension. Lifestyle modification is the first-line antihypertensive treatment. In addition, changes in lifestyle can increase the effects of antihypertensive treatment [37].

Weight gain and obesity are important risk factors for hypertension. Therefore, keeping weight under control and avoiding obesity, particularly abdominal obesity, are essential for controlling and preventing blood pressure. A ratio of waist circumference to height of less than 0.5 is recommended for most populations [38].

In a meta-analysis study including 25 clinical trials and 4,874 participants, the results showed that for every kilogram of weight loss caused by energy consumption restriction, physical activity, or both, the average SBP and DBP reductions were 1.05 mmHg and 0.92 mmHg, respectively. The effect of weight loss on DBP was significantly greater in the population taking antihypertensive drugs than in the untreated population (5.31 mmHg vs. 2.91 mmHg). This meta-analysis clearly demonstrated that weight loss was essential for both the prevention and treatment of hypertension [39].

There is strong evidence supporting the existence of a link between high salt intake and hypertension.

The results of a meta-analysis study in China showed that salt substitution led to a significant reduction in the average SBP (-5.7 mmHg) and DBP (-2 mmHg). Moreover, teaching to reduce salt consumption in schools led to a decrease in parents' SBP (-2.3 mmHg) [40].

The results of another meta-analysis on 85 RCT with dose–response analysis of sodium reduction showed an approximately linear relationship between sodium intake and reduction in both systolic and diastolic BP across the entire range of dietary sodium exposure. Although this occurred independently of baseline BP, the effect of sodium reduction on level of BP was more pronounced in participants with a higher BP level [41].

Regarding the effect of diet, the results of a meta-analysis of clinical trial studies revealed that adherence to the Mediterranean diet led to a decrease in the average rates of SBP and DBP by 2.35 mg and 1.58 mmHg, respectively [42].

The findings of studies indicate that regular aerobic and resistance exercise may be beneficial for both the prevention and treatment of hypertension. The results of a meta-analysis study including 11 studies on antihypertensive drug users and 25 studies on non-antihypertensive drug users showed that resistance exercise in antihypertensive drug users led to a reduction in SBP (from 1.6 to 2.8 mmHg) and DBP (from 4.6 to 1.6 mmHg). Muscle strength increased significantly in both groups of users and non-users of antihypertensive drugs [43]. The results of a systematic review study also showed that performing tai chi movements, which is originally a Chinese exercise and a combination of deep breathing relaxation techniques and gentle conscious movements, can be recommended as an auxiliary treatment to control hypertension, especially for patients younger than 50 years old [44].

Smoking causes a sharp increase in blood pressure and heart rate. In a clinical trial study in smoking hypertensive patients, by Holter monitoring of blood pressure every 30 min during 24 h, patients' blood pressure and heart rate were evaluated on a day when they smoked and, on another day when they did not. The results showed that the 24-h average blood pressure, daytime blood pressure, and heart rate were significantly higher during the smoking period than in the non-smoking period [45].

Excessive alcohol consumption raises the risk of CVDs, such as cardiomyopathy, hypertension, atrial arrhythmia, or stroke. The findings of a meta-analysis study including 15 clinical trial studies and 2,234 participants revealed that reducing alcohol consumption led to a significant decrease in average SBP by 3.31 mmHg and a drop in average DBP by 2.04 mmHg. A dose–response relationship was observed between the average percent reduction in alcohol consumption and the average blood pressure reduction [46]. On the other hand, the result of another study demonstrated that in spite of consuming less alcohol by patients with hypertension, there was no change in SBP, DBP, and outcomes, such as overall mortality, mortality due to cardiovascular events, and cardiovascular events, compared to the control group [47].

Teaching self-care behaviors is an important approach for blood pressure management and control. The results of a meta-analysis study of 18 clinical trials showed that self-care activities could lead to the management and treatment of hypertension. Such measures can also reduce emergency and outpatient visits by 40% and 17%, respectively [48]. The overall reliability of the presented evidence was evaluated as high.

### Evidence-to-decision considerations

According to the findings of a qualitative survey on the values and preferences of hypertensive patients, they preferred lifestyle modification to medication. Additionally, patients considered diet adherence, physical activity, stress control, and the acceptance of the disease to be effective factors in controlling and treating blood pressure, and they requested more education regarding lifestyle modification to manage their blood pressure [49].

The members of the GUG estimated the resources and costs required to implement recommendations related to lifestyle modification to be small. A huge information gap exists in this regard, and Quality cost-effectiveness studies were not available in this area [50].

The recommendations provided by the members of the GUG were acceptable to the key beneficiaries and were implementable. The results of our qualitative survey on general practitioners indicated that regarding treatment, more attention is paid to drug treatment and less to lifestyle, and appropriate referrals in this field (e.g., referral to a nutritionist, sports medicine specialist, and psychologist or healthy lifestyle consultant) are not observed, an issue that needs to be taken into account [30].

**Box 5:** Recommendation and Suggestion for Threshold to start Pharmacological treatments

1- It is recommended that the threshold for starting pharmacotherapy in people under 75 years of age with hypertension and without comorbidities, be considered as SBP ≥ 140 mmHg and/or DBP ≥ 90 mmHg 2- It is suggested that the threshold for starting pharmacotherapy in individuals over 75 years of age, should be SBP ≥ 150 mmHg and/or DBP ≥ 90 mmHg 3- It is recommended that in individuals with CVD, CKD (who have albuminuria more than 30 mg/g Cr), and diabetes mellitus, the threshold for starting pharmacotherapy should be SBP ≥ 130 mmHg and/or DBP ≥ 80 mmHg 4- It is suggested that in people with a history of stroke, the threshold for starting pharmacotherapy should be SBP ≥ 130 mmHg and/or DBP ≥ 80 mmHg	

### Evidence and rationale

Based on the results of a meta-analysis of 123 studies with 613,815 participants, every 10-mmHg reduction in SBP significantly decreased the risk of major CVD by 17–23%, coronary artery disease by 12–22%, stroke by 23–33%, HF by 22–33%, and death due to all causes by 9–13% in the studied populations [50].

A meta-analysis study of 48 clinical trials with 344,716 participants showed that a reduction of 5 mmHg in SBP, regardless of previous diagnoses of CVD, and even in normal or high blood pressure values, decreased the risk of MACE by approximately 10% [51]. The overall reliability of the evidence was high, and the results of related studies showed that the predicted benefits of lowering blood pressure (SBP < 140 mmHg in the general population and < 130 mmHg in the high-risk population) included a reduction in mortality, cardiovascular mortality, strokes, MIs, and HF events. The predicted harms were mostly non-serious adverse events, and some were surrogate outcomes, such as increased creatinine [51–59].

### Evidence-to-decision considerations

The results of our qualitative study demonstrated that patients' understanding of hypertension was often limited and they were not aware of the existence of treatment options; however, counseling provided opportunities for patients and physicians to reach a common understanding of their treatment choices [60].

From the point of view of the panel members, the resources and costs required to start drug treatment at the mentioned threshold were evaluated as intermediate. A qualitative study's findings revealed that the factors related to health systems, such as the lack of specialists, the cost of medicine, the length of a visit session in the clinic, and the possible weak services provided, were among the major barriers preventing patients from visiting the clinic and starting treatment. In developing countries, weak health systems have been identified as a major obstacle in effectively addressing the growing burden of chronic conditions, such as hypertension [61].

Epidemiological studies have shown that the benefits of treatment (with both lifestyle interventions and medications) clearly outweigh the risks of treatment [62]. Furthermore, modeling studies support the effectiveness and cost-effectiveness of treating younger and low-risk patients [22].

Due to the lack of evidence on the impact of the mentioned threshold on health inequality, this impact was considered unclear by the panel members [63]. From the perspective of the panel members, initiating pharmacotherapy at the mentioned threshold was acceptable to the key beneficiaries and was applicable.

**Box 6:** Recommendation and Suggestion for first-line monotherapy of hypertension

1- It is recommended for people with hypertension and without comorbidities who need pharmacotherapy, the first-line Medicine should be one of antihypertensive drugs that consists of angiotensin-converting enzyme inhibitors (ACEIs) or angiotensin receptor blockers (ARBs), thiazide and thiazide-like agents, and calcium channel blockers (CCBs) 2- It is recommended that in people over 75 years of age with hypertension, the first medicinal treatment should be the use of one of the CCBs or thiazide and thiazide-like agents 3- It is recommended to use one of the medicines in the class of ACEIs/ARBs, CCBs, or thiazide and thiazide-like agents in patients with hypertension and diabetes mellitus (without albuminuria) 4- It is recommended that in patients suffering from hypertension and CKD (who have albuminuria more than 30 mg/g Cr), the first-line therapy should be one of the medications in the medication class of ACEIs or ARB 5- It is recommended that in patients with hypertension and a history of stroke, the first pharmaceutical treatment should be the use of one of the medications in the medication class of ACEIs/ARBs, thiazide and thiazide-like agents, and if not effective or side effects occure, one of the CCB drugs should be used 6- It is recommended to use one of ACEIs or ARBs or beta blockers in patients with hypertension and coronary artery diseases 7- It is suggested to use one of the long-acting CCB drugs in patients with hypertension and coronary artery disease if beta-blockers are not effective or are contraindicated 8- It is recommended that in patients suffering from hypertension and HF, the first-line treatment should be one of the ACEIs or ARBs drugs	

### Evidence and rationale

Data from 32 systematic reviews to obtain evidence on the benefits and harms of different medication classes revealed various benefits, including a reduction in mortality and MACE, per 1,000 people undergoing treatment with different medication classes [64].

The results of a systematic review comparing CCBs with other medication classes in blood pressure management, which included the review of 23 clinical trial studies with 153,849 participants having hypertension, showed that there was no difference in death due to any cause between CCBs as the first-line treatment and other antihypertensive medication classes. It is probable that CCBs had increased the incidence of MACE and CHF-related events in comparison to diuretics (moderate- certainty evidence). It was found that compared to beta-blockers, CCBs led to a reduction in the outcomes of MACE, stroke (moderate-certainty evidence), and cardiovascular death (low-certainty evidence). CCBs decrease stroke risk (low certainty) and increase CHF risk when compared to ACEIs. They also reduce the risk of MI (moderate-certainty evidence) and increase the risk of CHF (low-certainty evidence) in comparison to ARBs. Overall, for the treatment of hypertension, there is moderate-certainty evidence indicating that diuretics lead to a reduction in the risk of MACE and CHF more than CCBs. Low-to-moderate-certainty evidence was obtained showing that CCBs were more likely than beta-blockers to reduce MACE, and similar level of evidence suggested that CCBs reduced the risk of stroke compared to ACEIs, and decreased the risk of MI compared to ARBs; however, they increased the possibility of CHF compared to ACEIs and ARBs [65]. A systematic review and network meta-analysis, in which the results of 15 observational studies and 7 clinical trials on 649,790 patients with hypertension were reviewed, showed that in observational studies, treatment with CCBs or ARBs was associated with a lower risk of dementia, in comparison to treatment with other medication classes [66].

In a systematic review of 44 clinical trials with 5,745 participants, aldosterone antagonists had unclear effects on renal failure, mortality, and cardiovascular events, in comparison to placebo or standard care. Aldosterone antagonists, compared to placebo or standard care, may decrease protein excretion, estimated glomerular filtration rate (eGFR), and SBP, and possibly increase the risk of hyperkalemia, acute kidney injury, and gynecomastia [67].

In subgroups, for patients over 65 years of age, the results of studies suggest usefulness of diuretics or CCBs. Diuretics are probably the most effective and CCBs the least effective drugs for HF prevention [68, 69].

Epidemiological studies showed that the benefits of treatment (either with lifestyle interventions or with drugs) clearly outweighed the risks of treatment [70]. Side effects of CCBs, ACEIs/ARBs, and thiazides were rare and usually mild and manageable [71, 72].

### Evidence-to-decision considerations

Most patients, healthcare workers, professional associations, and government organizations are fully aware of the importance of drug therapy for hypertension treatment in preventing multi-organ complications. However, asymptomatic patients may consider treatment of little value, unless they have been convinced that there is a relationship between the immediate discomfort/side effects of the drug and potential long-term health gains [17]. In our qualitative study, hypertensive patients preferred the prescription of medicine by a specialist over a general practitioner and believed that taking medicine more often effective in the treatment and would have fewer adverse effects. On the one hand, from health care providers' viewpoint, patient compliance is crucially important because the disease is chronic and the patient has to take the medicine for long time; nevertheless, it is challenging to convince patients that blood pressure medication should be continued even with blood pressure regulation, a fact that is not easily accepted by them [49]. The results of this qualitative study showed that numerous individual and social factors were effective on adherence to treatment, among which is the lack of family support and the need for separate meals to improve treatment as individual factors, and the feeling of security, local facilities, and drugs availability as environmental factors [17].

Regarding the use of resources and costs of treatment, the members of the GUG estimated them to be small due to the low price of antihypertensive drugs in the country and the fact that these drugs are covered by insurance. The results of a study showed that treatment for stage 1 hypertension was cost-effective for all men and women between the ages of 45 and 74 [73].

From the perspective of the panel members, the mentioned pharmaceutical treatments were acceptable to the key beneficiaries and were implementable. Our qualitative study on physician’s findings showed that they believe although there were several medication classes in international guidelines, it was not clear for them which one to follow and what drug needed to be prescribed as the first line of pharmacological treatment. On the other hand, some recommended drugs are not available or are expensive in our country, therefore, the national guidelines need to specify the drugs that are the most efficient and reasonably priced in different steps [30].

**Box 7:** Recommendation and Suggestion for first-line combined drug therapy of hypertension

1- It is recommended for people with hypertension who prescribed a combination pill of different classes of antihypertensive drugs, as the first-line treatment, a combination pill including ACEIs/ARBs, CCBs, diuretics (thiazide/thiazide like diuretics), to be used to increase compliance and adherence to treatment 2- In patients with hypertension and diabetes mellitus who need drug therapy, a combination therapy of ACEIs/ARBs and CCBs or ARBs/ACEIs and thiazide/thiazide like diuretics is recommended 3- In patients with hypertension and a history of stroke, a combination therapy of ACEIs/ARBs and thiazide/thiazide like diuretics or ACEIs/ARBs and CCBs is suggested 4- In patients with hypertension and coronary artery diseases, a combination therapy should be recommended based on the patient's condition using ACEIs/ARBs and beta blockers or ACEIs/ARBs and CCBs 5- It is recommended that for patients suffering from hypertension and HF without a reduction of ejection fraction, a combination therapy of one of the ACEIs/ARBs drugs and beta blockers be used 6- In hypertensive patients whose DBP is high with or without SBP, it is suggested to adopt a combination therapy using ACEIs/ARBs and CCBs or ACEIs/ARBs and diuretic	

### Evidence and rationale

The latest hypertension guidelines have increasingly emphasized the use of combination therapy as the first-line treatment in various patients [10, 11]. Unlike previous guidelines that traditionally recommended a stepped care strategy with initial monotherapy [62], this shift in emphasis on combination therapy as the first-line treatment is reflective of several factors. Most patients with hypertension need to be treated with two or more medications to reach the target blood pressure; however, many of them do not receive such treatment. There are concerns about the risks associated with long-term hypertension control and that the need for multiple clinic visits may negatively affect long-term adherence [74, 75]. These factors have intensified clinical interest in the employment of combination therapy as the primary therapy. Nevertheless, concerns remain about the evidence base to support this strategy, particularly in relation to the risk of adverse events.

The findings of a systematic review including 33 clinical trials with 13,095 participants demonstrated that the use of low-to-standard dose combination therapy compared to standard monotherapy led to the effective control of hypertension without withdrawal from treatment due to adverse effects [76]. Examining 568 patients in a review study, it was found that combination therapy, compared to monotherapy, was associated with a decrease in total mortality (0.8 to 21.72), mortality caused by CVDs, cardiovascular events (0.22 to 4.41), Serious side effects (0.31 to 1.92), and withdrawal due to side effects (0.53 to 1.35) [77].

A systematic review compared single-pill combination therapy containing azilsartan medoxomil and chlorthalidone with the simultaneous administration of two tablets of azilsartan medoxomil and chlorthalidone. The results indicated a greater decrease in SBP in the single-pill combination therapy group (35.1 mmHg) than in the simultaneous administration of two tablets (29.5 mmHg). The findings of another study on 153 individuals with CKD showed that the use of a single-pill combination therapy including azilsartan medoxomil and chlorthalidone, compared to other single- pill combination therapies, led to a more effective reduction of blood pressure and increased adherence to treatment [78].

As a result of a clinical trial involving 125,635 patients, researchers found that two-drug combination therapy in the form of a single tablet or as a separate medication reduced the risk of death by 11–28% and hospitalization due to heart complications by 10–21% compared to single-drug therapy (10–21%) [79].

The overall certainty of the evidence found was moderate. Despite the favorable effects of combination therapy, which include improved adherence and continuation of treatment, and consequently, improved blood pressure control and better clinical outcomes, there have been reports of a few adverse effects, such as the side effects of drugs, especially when drugs are combined in one tablet.

### Evidence-to-decision considerations

Our qualitative survey of patients' values and preferences revealed that they preferred drugs with fewer side effects [49]. The results of a systematic review study showed that simplifying the medication regimen of patients led to an increase in their treatment compliance from 6 to 20% [80].

The resources and costs required for the combination therapy were evaluated as moderate by the members of the GUG. In a meta-analysis study, it was found that patients taking single-pill combination therapy had higher total annual healthcare costs than those treated with compounded medications [81]. Although combination therapy is initially associated with a moderate increase in required resources and costs, such as direct drug costs and manufacture and supply chain costs, the net profit of improving blood pressure control and reducing major events related to the hypertensive process outweighs the increased cost because the control of hypertension is probably achieved earlier with combination therapy.

Due to the lack of evidence, the effect of combination therapy on health inequalities was assessed as unclear by the members of the GUG. Moreover, according to them, the mentioned combination therapies were acceptable to the key beneficiaries and were implementable. In a qualitative interview, physicians stated that different times and conditions were provided in the guidelines for prescribing one or more drugs or compounded medications; therefore, there was a need for explicit instruction in this regard. Furthermore, the polypharmacy and different brand names of medications led to confusion in patients and sometimes to their non-compliance with prescribed treatment [49].

**Box 8:** Recommendation and Suggestion for visit intervals before and after reaching the treatment goal of hypertension

1- It is suggested that at starting the drug therapy or in case of any change in the type or dosage of antihypertensive drugs, the visit sessions should be held on monthly intervals until reaching the treatment goal 2- It is suggested to check the patient every 3 to 6 months after reaching the treatment goal	

### Evidence and rationale

The SR group did not find strong recent evidence on the appropriate visit intervals in patients with hypertension before and after reaching the treatment goal. However, there were older studies reported in this field. In a clinical trial study, the follow-up intervals of 3 months and 6 months were compared with each other in patients aged 30 to 74 years with hypertension. The results showed that the average blood pressure, blood pressure control level, patient satisfaction, and treatment adherence did not differ between 3- and 6-month follow-up intervals [82]. Based on the findings of another clinical trial study, follow-ups with shorter intervals would lead to more predicted favorable outcomes, such as better blood pressure control and monitoring of side effects, and perhaps improved adherence, but may be associated with higher costs. Therefore, longer follow-up periods are inevitably expected to cause harm [79]. In large studies such as ACCORD and SPRINT, which showed significant improvement in cardiovascular events with blood pressure control, the initial follow-up period was one month [83, 84]. Undesirable outcomes of holding follow-up sessions with shorter time intervals would increase the treatment burden on patients and the country's health system.

### Evidence-to-decision considerations

No evidence was found for patients' values and preferences regarding the proper intervals for patient visits. Re-evaluation of hypertensive patients over 65 years of age or patients who live alone in shorter time intervals, in addition to faster identification of clinical complications, builds confidence in patients and provides them security; however, in younger and especially asymptomatic patients, visits in short intervals may interfere with their family and work responsibilities [85].

There is no evidence for the costs, resources, and cost-effectiveness of reducing or increasing follow- up intervals; nevertheless, it seems that decreasing intervals would be cost-effective in the elderly, and the application of telemedicine methods for follow-up would be helpful as well [86]. The effect of the suggested proposals is unclear on health inequalities.

The members of the GUG also assessed the acceptability of intervals provided by key beneficiaries as unclear., since patients' adherence to treatment was highly important but was not easily accepted by patients, following up with patients was crucial [30]. The GUG members reached a consensus on an appropriate interval that were suggested for visiting patients under pharmacotherapy before and after reaching the treatment goal.

**Box 9:** Suggestion for Minimum tests required to initiate and continue treatment of Hypertension

1- It is suggested to examine serum electrolytes, creatinine, lipid profile, fasting sugar, urinalysis, and electrocardiogram before starting pharmacotherapy in people with hypertension 2- It is suggested that during treatment, laboratory tests be performed according to the patient's conditions and the appropriate time intervals for these tests to be determined based on the same criterion. In patients who lack comorbidities, tests should be performed annually	

### Evidence and rationale

Before starting pharmacotherapy and to achieve the treatment goal, it is necessary to carry out tests to identify cases of secondary hypertension (in case it was overlooked before) and to detect comorbidities like diabetes mellitus, dyslipidemia damage to lower extremities, and possible side effects of treatments (e.g., high uric acid and abnormal electrolytes), as well as to assess the risk of CVDs, cure other risk factors, and choose appropriate antihypertensive drugs [86].

Performing preliminary tests before initiating the treatment may cause a delay in the onset of the treatment and the possible occurrence of cardiovascular events, therefore, treatment should be started earlier and without delay.

### Evidence-to-decision considerations

Concerning patients' values and preferences for conducting tests at the beginning and during hypertension treatment, the members of the GUG believed that there was probably no important uncertainty. However, patients believed that some tests were costly, which sometimes prevents the performance of tests [30]. The results of a cohort study showed that the frequency of doing elderly patients' tests who were recently treated with antihypertensive drugs was lower than expected. Accordingly, 59% of patients had not performed any tests before starting antihypertensive therapy, and at a follow-up with a mean of 10 months, less than half of them had tests done, and nearly two- thirds of them had not had their serum electrolytes or renal function tested even once [87].

The resources and costs required to carry out the recommendations were evaluated as average. The cost of conducting tests for a person is relatively negligible compared to the overall costs of lifelong treatment and complications. Nonetheless, performing tests will have a significant impact on the health systems due to the high prevalence of blood pressure in most societies [88]. In countries with fewer resources, the costs of testing may affect its performance. In regions with plenty of resources, laboratory tests are simple to conduct; however, where resources are scarce, having to conduct such tests before treatment onset can hinder treatment and foster inequality [89]. The recommendations presented were acceptable to the beneficiaries and were implementable.

**Box 10:** Recommendation and Suggestion for Telehealth strategies to reduce subsequent complications of hypertension

1- It is suggested that employees of health care units use Telehealth strategies for BP treatment and control, such as creating electronic health records, training the use of mobile phone applications, and sending text and video messages 2- In order to monitor and record blood pressure, it is suggested that patients use Telehealth strategies, such as mobile phone applications	

### Evidence and rationale

Tele Health strategies, such as Telemedicine, eHealth, and mobile-based technologies (e.g., mobile health [m Health]), are new tools that facilitate the process of controlling patients with hypertension [90]. A telemedicine-based intervention can decrease the level of SBP and DBP during the day in populations suffering from hypertension [91]. However, this drop is influenced by various factors, such as behavioral goals, intervention components, implementation methods, and patient participation [90, 92]. Moreover, noticeable outcomes are associated with the role of social networks, social media, and electronic technology as practicable components of lifestyle modification and disease management programs [92].

Although m Health interventions are generally promising in reducing SBP in hypertensive patients, results are inconsistent and it is unclear which combination of telehealth intervention features is most effective; furthermore, there is no sufficient evidence that telehealth, as an independent strategy, can effectively control blood pressure [93–96].

Good blood pressure management is a multifactorial process and requires the participation of patients, families, health care providers and systems. Implementing strong improvement strategies can be successful in identifying and controlling blood pressure levels [97, 98]. On the other hand, studies have shown that for patients with a stable condition, home blood pressure monitoring and electronic communications with the doctor may provide an acceptable alternative to reduce the frequency of visits [82]. Therefore, such strategies as electronic health records and telephone and electronic follow-ups are recommended.

### Evidence-to-decision considerations

Examples of eHealth include mobile and wireless technologies, health information technology, remote medicine (telemedicine), health management, and health-related education [99]. Based on the opinion and experience of the members of the GUG, the use of telehealth strategies is necessary to control chronic diseases and they are accessible and easy to use. Examining the values and preferences of the patients also showed that, from the patients' point of view, these technologies are a desirable method for educating and following up with patients [49].

The overall certainty of the evidence found for the positive effect of employing telehealth strategies on blood pressure control was high, and numerous positive effects, such as improving people's health, and boosting the quality of life, have been reported for all types of these strategies, including mHealth, telemedicine, and eHealth [99–101].

Regarding costs and resource consumption, a meta-analysis study showed that effective eHealth interventions should target large populations since they impose limited additional costs. These interventions should involve a lot of participants, and eHealth should be employed as an alternative to routine care [102]. Regarding this, the required costs and resources were considered low by the GUG members.

Due to the lack of sufficient evidence, GUG believed that the effect of these interventions on health inequalities was unclear, and that this intervention was acceptable to the key beneficiaries and was implementable because its infrastructure was available in the country.

**Fig. 2:**
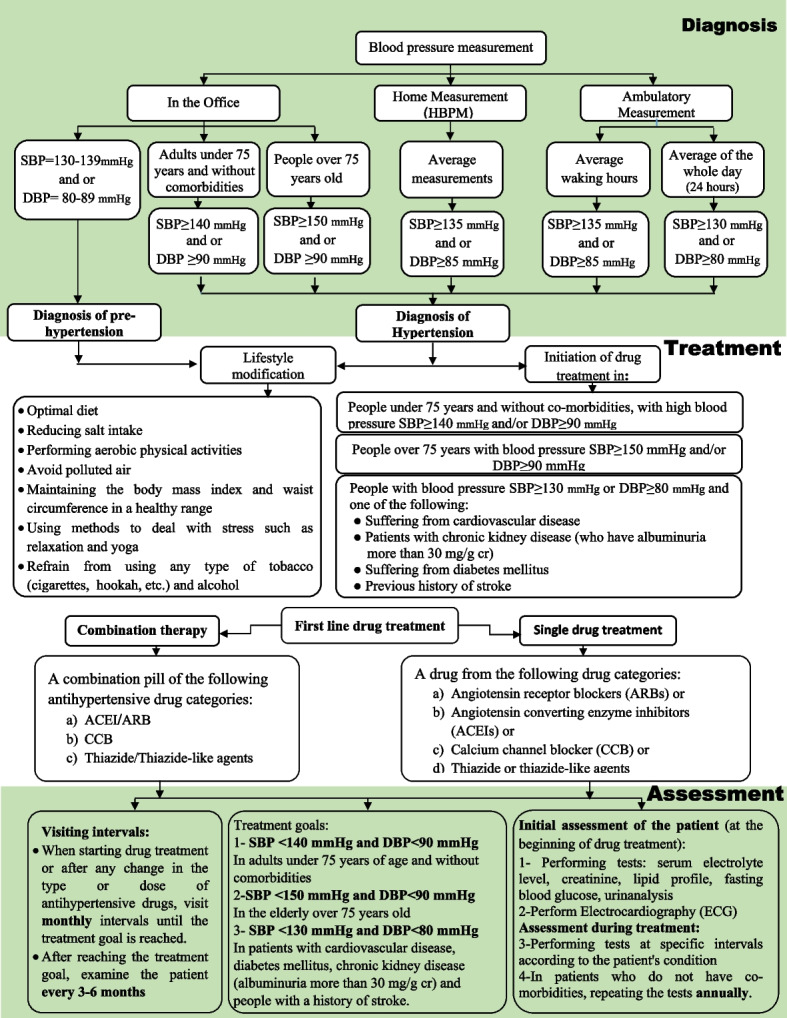
The Flowchart of the Diagnosis and Treatment of Hypertension in Adults (Summary of Guideline Recommendations)

In general, the GUG members, taking into account the results of the previous studies and their clinical experiences, extracted significant key points that needed to be considered in the management of patients with hypertension, which are presented in Table 1. Moreover, the algorithm for diagnosis, management, and treatment of hypertension was drawn based on the recommendations and suggestions of the updated guideline, which is illustrated in Fig. 2. This figure contains all the content presented in the recommendations and suggestions section in a summary form.

**Table 1:**
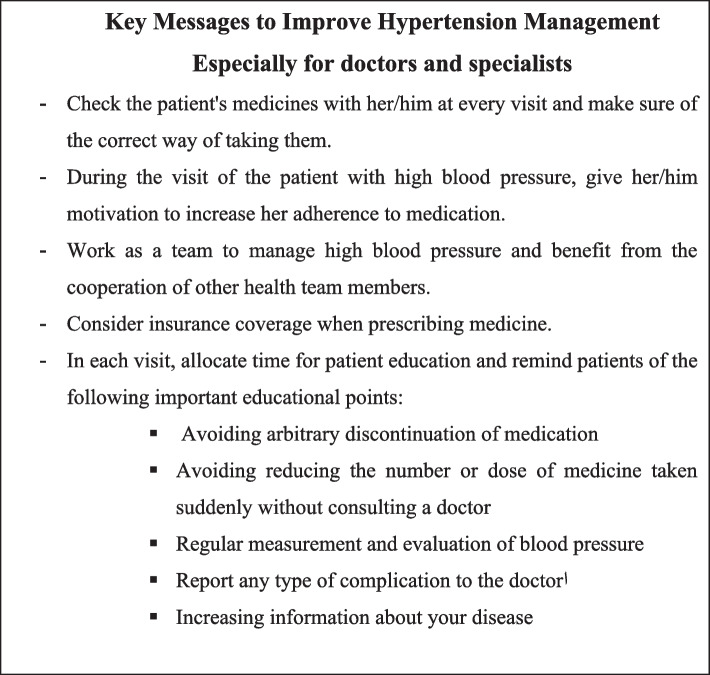
Key messages to improve hypertension management

## Discussion

Despite significant progress in diagnosis and treatment of hypertension, it is still the most important cause of death from cardiovascular diseases in Iran. In this study, the latest national guideline for hypertension diagnosis, management, and treatment was updated by a group of experts in different but related fields from multiple universities of medical sciences and scientific associations or societies who consist the GUG. They reviewed and updated the scope of previous version of the guideline, including the functional area, target groups, outcomes, and PICO questions, and according to the changes applied in all these sections, they did a comprehensive update.

Participating groups that involved a wide range of health care providers (i.e., specialists, general practitioners, nurses, nutritionist, epidemiologist and health economists) and patients, tried to address the issues in disease management that had not been dealt with in the previous version of the guideline through active participation and interaction, and provide, as much as possible, practical and applicable recommendations and suggestions to resolve them.

In updating of this guideline, an attempt was made to take into account the new developments that have occurred in the management and treatment of hypertension, especially drug treatment and technical interventions. Also, the users of the previous versions of the guideline were only physicians, and other members of the health team were not considered, but the users of the updated version are all PHC health care system employees and patients and their families. In this guideline, a simple and practical treatment algorithm was designed for users. It is also suggested that a mobile application be designed based on guidelines for physicians and health care providers as well as patients and their families in order to apply the recommendations and suggestions of the guideline in the daily management of hypertension.

## Conclusion

The authors believe that this guideline, in addition to highly benefitting service providers involved in the diagnosis, treatment, and management of hypertension, will be of great benefit to patients and their families too. It is because this guideline was prepared by employing all standard steps required, using the latest available scientific evidence, and based on the existing infrastructure in the country. Therefore, it is suggested that this guideline be provided to the Ministry of Health to be distributed to all, scientific associations, health centers and followed by the health care providers in the public and private sectors.

## Data Availability

Not applicable.

## Abbreviations

ACEI: Angiotensin-converting enzyme inhibitors
ARBs: Angiotensin receptor blockers
ABPM: Ambulatory Blood Pressure Monitoring
BP: Blood Pressure
CCBs: Calcium Channel Blockers
CVD: Cardiovascular Disease
CKD: Chronic Kidney Disease
CR: Creatinine
DM: Diabetes Mellitus
EF: Ejection Fraction
ERG: External Review Group
FGD: Focus Group Discussion
GUG: Guideline Updating Group
HBPM: Home Blood Pressure Monitoring
mg/gCr: Milligram Per Gram Creatinine
PICO: Population, Interventions, Control, Outcome
SC: Steering Committee
SRG: Systematic Review Group
